# The Frequency Response of the Vibrissae of Harp Seal, *Pagophilus Groenlandicus*, to Sound in Air and Water

**DOI:** 10.1371/journal.pone.0054876

**Published:** 2013-01-22

**Authors:** Lisa F. Shatz, Theodorus De Groot

**Affiliations:** 1 Electrical and Computer Engineering Department, Suffolk University, Boston, Massachusetts, United States of America; 2 Department of Biomedical Engineering, University of Wisconsin-Madison, Madison, Wisconsin, United States of America; University of Zurich, Switzerland

## Abstract

The motion of isolated seal vibrissae due to low frequency sound in air has been measured using a microscope with a video camera and modeled using an FEM method with good agreement between the measurements and the model; the model has also been used to predict the motion of seal vibrissae in water. The shape of the seal vibrissae is that of a tapered right rectangular prism, unlike that of the previously studied rat vibrissae which are conical in shape. Moreover, unlike rat vibrissae which oscillate in the direction of the sound stimulus, two different modes of vibration of seal vibrissae were observed – one corresponding to the wider side being stimulated and one with the narrow side stimulated. The tuning of the seal vibrissae was much sharper than those of rat vibrissae, with quality factors about three times as large as those of rat vibrissae. As shown by the model, this increased sharpness is caused by the larger cross-sectional areas (by more than a factor of ten) of the seal vibrissae. This increased sharpness may be necessary for seal vibrissae so that they can have tuning in water, where the drag more heavily dampens the tuning than in air. The results suggest that vibrissae tuning may be important in the seal's ability to track the wake of its prey.

## Introduction

Vibrissae or whiskers play a crucial role in detecting disturbances in their surrounding fluid. Rat vibrissae have been shown to be frequency selective in their response to tactile stimulation [1], [2], and to sound stimulation [3]. Frequency selectivity means that each vibrissa has a frequency or range of frequencies to which it responds best. Shorter vibrissae have been shown to respond best to higher frequencies while longer vibrissae respond best to lower frequencies.

Pinnipeds, the taxonomic group that includes seals, sea lions and walruses, have the ability to use their vibrissae for tactile sensing [4]–[6], to track water waves as small as a micron in amplitude left in the wake of their prey [7], and to distinguish different wake patterns [8].

A 2011 study of the response of pinniped vibrissae to fluid flow [9] indicated that the isolated vibrissae of harbor seals (Phocidae) and California sea lions (Otariidae) were able to detect vortex shedding frequencies (3–7 Hz) produced in the wake of a stationary immersed cylinders in rotational fluid flows. Spectral analysis showed that the vibrissae were frequency-selective, and the dominant frequency of each vibrissa depended on flow speed. The relationship between vibrissae length and frequency selectivity, however, was not studied.

This paper presents a study of the mechanics of harp seal, *Pagophilus Groenlandicus*, vibrissae motion. It describes measurements and model predictions of how seal vibrissae respond to sound in air and models predictions of the response of seal vibrissae to sound in water. Its purpose is to determine the frequency selectivity of seal vibrissae in order to further understanding in how seals might use their vibrissae in sensing. The model gives good predictions for the motion of vibrissae. The same model, using the properties of water, was used to predict the motion of the vibrissae in water.

This work has applications for designing sound sensors, for understanding the neural responses of vibrissa sensing, and will serve to further study in vibrissae of aquatic mammals.

## Materials and Methods

**Figure 1:**
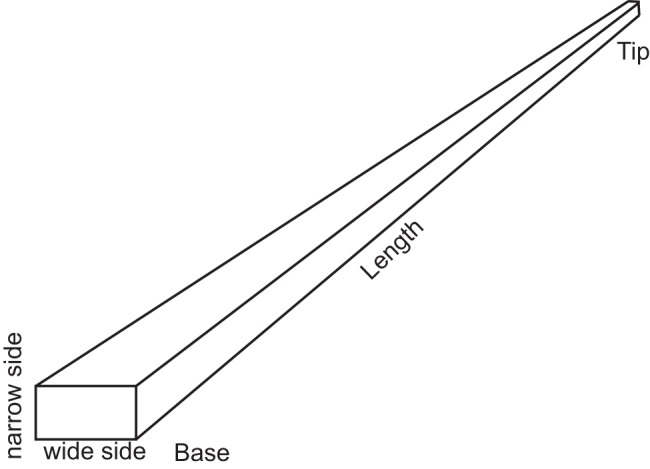
Model of vibrissa.

**Figure 2:**
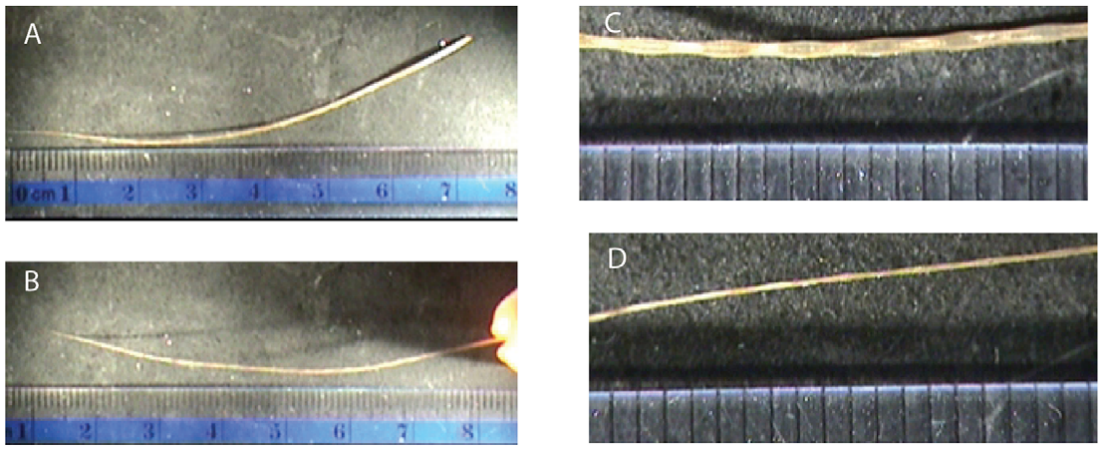
An 8 cm long vibrissa. (A): Wide side facing camera; (B): Thin side facing camera; (C): Close up of wide side in the midsection of the vibrissa; (D) Close up of thin side in the midsection of the vibrissa. Each ruler division is 1 mm wide. Although undulations were observed (see inset (C)), these were not modeled.

Vibrissae were plucked from a patch of face from a harp seal cadaver that was obtained from the New England Aquarium. The shapes of the vibrissae were seen to be that of a tapered right rectangular prism (Figure 1, Figure 2) with small undulations. The lengths of the vibrissae were measured with a ruler and the widths of the vibrissae, were measured at 1 cm intervals along the length of the vibrissae with Mitutoyo™ micrometers which are accurate to 1 μm. The masses of the vibrissae were measured with A&D HR-120 and A&D ER-120A balances accurate to 10 μg. From these measurements, the mass densities of the vibrissae were computed. The mean mass density for the 15 vibrissae that were used in this study was found to be 911 kg/m^3^ with a standard deviation of 100 kg/m^3^. The range of lengths of vibrissae on the harp seal face was found to be from .8 cm- 10.5 cm. The widths are on the order of tenths of millimeters, with the wide side about a factor to two longer than the thin side and the widths at the base about a factor of four larger than at the tip.

**Figure 3:**
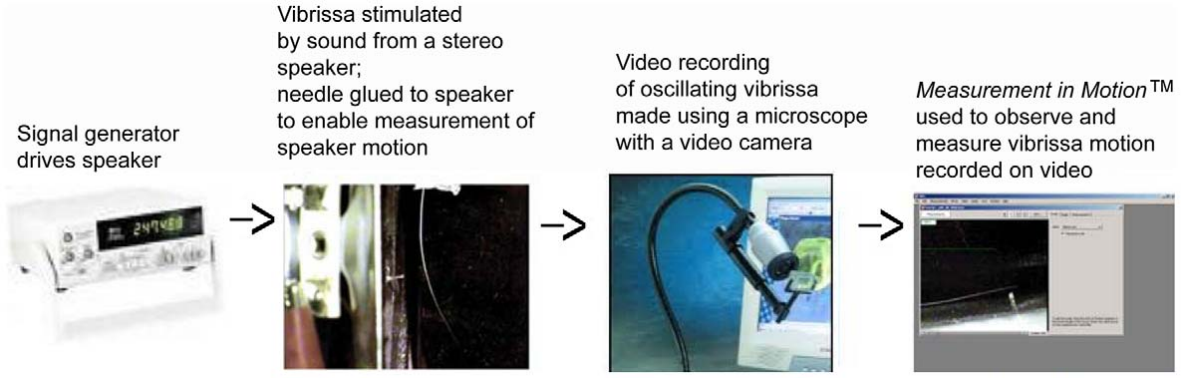
A block diagram describing the experimental set-up.

**Figure 4:**
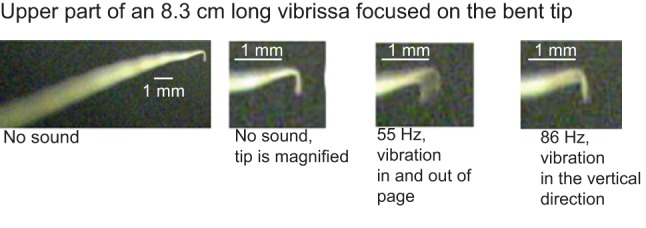
Motion of an 8.3 cm long vibrissa with the camera in front of the vibrissa tip, with the sound off, and at the two fundamental frequencies for the two vibration modes. The speaker is facing the length of the vibrissa (i.e. facing the page). The left two panels show the same case, the vibrissa with the sound off – the camera is focused on the tip, so that the length of the vibrissa is blurred. Although the wider side of the base of the vibrissa faces the speaker, because the vibrissa twists and curves, the wider side does not directly face the speaker throughout the length of the vibrissa and so both modes are excited. It can be seen by looking at the tip that the motion for 55 Hz (mainly in the direction coming in and out of the page, corresponding to the wider side of the vibrissa being stimulated) is perpendicular to the motion at 86 Hz (mainly in the vertical direction, corresponding to the thinner side of the vibrissa being stimulated).

The experimental set-up in which the vibrissae motion was recorded is shown in Figure 3 and is also described in a previous study [3]. The vibrissae are stimulated by the motion of the surrounding fluid (air) that is driven by the sound coming from the speaker. In this study a Weber™ 8 Ω 15 W speaker as the sound source was used with an Agilent E4432B signal generator through an InterM™ 120 W amplifier. Motion of the vibrissa was seen with a Ken-A-Vision VideoFlex Microscope Camera. A needle glued to the speaker surface allowed measurement of the speaker displacement which was used to approximate the air displacement that was exciting the vibrissa. As in a previous study [3] the speaker displacement was divided by two to approximate the air displacement in the vicinity of the vibrissa, since it had been determined, using a velocity microphone, that the amplitude of the disturbance decreased by roughly a factor of two from a position next to the speaker to one in the vicinity of the vibrissa. More details about approximating the attenuation can be found in that study [3]. Detecting a resonant frequency was done by incrementing the signal generator in 1 Hz intervals and observing the motion with the video camera. This detection was more difficult than in detecting the rat vibrissa resonance [3] because motion of the seal vibrissa was not necessarily in the same direction as the speaker motion. Because it is rectangular in cross-section, a seal vibrissa has two modes of motion; it can either move in the direction perpendicular to its wide side, or to its thin side, depending on the frequency. Because the vibrissae often twisted and curved (particularly the longer ones) either mode of motion could be stimulated (Figure 4). It can be seen in Figure 4 by looking at the tip that the motion for 55 Hz (mainly in the direction coming in and out of the page, leading to the apparent left to right motion in the projection, corresponding to the wider side of the vibrissa being stimulated) is perpendicular to the motion at 86 Hz (mainly in the vertical direction, corresponding to the thinner side of the vibrissa being stimulated). In order to better elucidate the mode of motion of the vibrissae, the experiment was done twice – once with the wider side of the base facing the speaker; and once with the thinner side of the base facing the speaker. The results for both types of stimulation were consistent; one or both modes were observed depending on which side faced the speaker, at the same frequencies, but the amplitude of motion for each mode changed with orientation. The flexible neck of the video camera allowed for viewing the motion of the vibrissae from different angles to better detect a resonance and measure the motion.

Measuring the vibrissa and speaker peak displacements was done by viewing a movie of the vibrissa and speaker oscillation, frame by frame, using the software *Measurement in Motion™*. The movie was taken at a frame rate of 27.97 frames per second with two images per frame. The camera was not synchronized to the speaker, but the speaker motion and the vibrissa motion were recorded simultaneously. The resonant frequencies were found by manually sweeping through frequencies and observing the output through the video microscope. The maximum displacements were recorded for each input frequency. The ratio of the vibrissa tip displacements to input displacement was plotted as a function of input frequency, and was compared to the same curve generated by a finite element model. Although the transduction occurs at the base of the vibrissa, the base motion would follow the tip motion. Only the fundamental frequencies of the two modes of motion were recorded. That the resonances were fundamental frequencies was clear from the vibrissa motion which did not have any nodes except at the clamped base. Higher order resonances were of such small amplitude that they were not consistently detected, and so were not recorded. Motion for vibrissae shorter than 3.3 cm was too small to be observed because their fundamental frequencies were highest and the speaker displacement decreases as the frequency increases. The purpose of the measurements is to determine the type of tuning each vibrissa has at its fundamental frequencies. Therefore, for each vibrissa, for each mode of motion, motion was recorded at its fundamental resonant frequency, at the highest and lowest frequencies for which motion was observable, and for points in between those frequencies, if the range wasn't too small (less than ∼10% of the resonant frequency).

### The FEM Model

#### Determining the Resonant Frequencies

As had been done previously [1]–[3], the vibrissae are modeled as thin elastic beams, although in this case, their shapes are that of right rectangular prisms. Equation (1) describes the motion of the vibrissae:
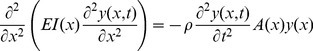
(1)
*y*(*x, t*) (m) represents displacements of the vibrissa as a function of *x*, the distance from the base; *E* (Pa) represents the vibrissa's Young's modulus; *I*(*x*) (m^4^) represents the second moment of area; ρ(kg/m^3^) represents the vibrissa's mass density; *A*(*x*) (m^2^), the cross-sectional area of the vibrissa. Because the vibrissa motion is small, we will approximate E as constant. Since the vibrissae can either be stimulated on their wide side, or on their narrow side, *I*(*x*) has two forms. When the wider side is stimulated:



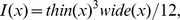
and when the narrow side is stimulated:



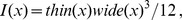
where *thin*(*x*) is the length of the narrow side at position *x* and *wide*(*x*) is the length of the wide side at position *x*. For sinusoidal motion *y*(*x, t*) can be represented as 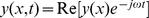 where ω represents the radian frequency of oscillation. Equation (1) can then be represented as,



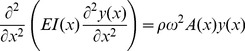
(2)As had been done in [1]–[3], we approximate the derivatives of *I*(*x*) as zero and represent Equation (2) as
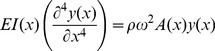
(3)


The solution for the eigenvalues of ω in Equation (3) are the resonant frequencies. These eigenvalues were found using a finite element method derived from a minimum energy formulation. Expressions for *thin*(*x*) and *wide*(*x*) were obtained by fitting a first, second, or third order polynomial to the measured widths along the length of the vibrissa. The lowest order expression that gave a coefficient of determination *r^2^* value of at least .94 was used.

The value for ρ was derived from the measurements of the masses and the dimensions of the vibrissae. The mean value of 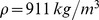 was used in each run of the model. This value is close to those measured for rat vibrissae, 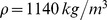 [2] and 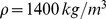 [1].

A cylindrical vibrissa was used to test how well resonant frequencies produced by the model matched with the resonant frequencies for a shape for which the exact resonant frequencies are known [10]. For a cylinder with length 3.6 cm and radius .26 mm, the maximum fractional error between the exact solution and the model's predictions for n = 19 elements was less than .000012.

### Determining the Motion of the Vibrissa in Fluid without Drag

To solve for the vibrissa displacement *y(x)* and expression for the fluid force must be added to Equation(3),
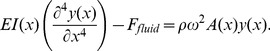
(4)


The same finite element model was used as before and, since the vibrissa is long and thin, the fluid force is represented by the solution for the force on an infinite oscillating cylinder in linear incompressible fluid [11]–[13], which has a known exact solution. The form of the solution that was used in this study is presented in a previous study [11] and will be given in the next section.

### Determining the Fluid Force

The fluid force per unit length of fluid oscillating perpendicular to a thin cylinder *F_fluid_* was first derived by Stokes [13] and reformulated by [12] and [11]. For sinusoidal motion the fluid velocity is given by 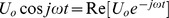 and *F_fluid_* is given by the real part of,
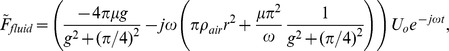
where r is the radius of the cylinder; μ μis the viscosity of the fluid; ρ_air_ is the mass density of the fluid; and g is given by



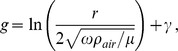
where the Euler's constant γ = .577.

The radius *r* of the cylinder used to model the fluid force on the vibrissa for each finite element is set equal to 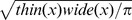 which decreases from base to tip. The previous study [3], which also used the approximate solution for the force on an oscillating spheroid in linear incompressible fluid to model the motion of the vibrissa, found no appreciable difference in the results between using the force from an oscillating cylinder or that from an oscillating spheroid, which would suggest that an approximate solution of the fluid force would give valid results.

### Determining the Motion of the Vibrissa in Fluid with Drag

The fluid force on the vibrissa is proportional to the difference in fluid velocity and the vibrissa velocity, and so we will separate the force into two terms: *F_fluid_* given in the previous section (proportional the fluid velocity) representing the drag on the hair in its equilibrium position and an additional drag force due to the small oscillations away from equilibrium which are proportional to the vibrissa velocity. As has been done in previous studies [3], [11], [12], the additional drag force is given by 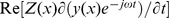, where 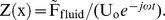 The additional drag term was introduced into Equation (4) giving Equation (5):
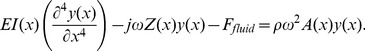
(5)


### The Finite Element Model

A finite element model using a minimum energy formulation was derived from Equation(5), and was used to solve for y(x). With the stiffness matrix [*kn*] corresponding to the 
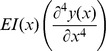
 term, the mass matrix [*mn*] corresponding to the 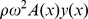 term, the force matrix [*tu*] deriving from F_fluid_, and the drag matrix [*tz*], deriving from Z(x), the vibrissa displacements [*yy*] can be obtained from the equations,
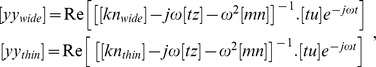
where [yy_wide_] and [kn_wide_] are the displacement and stiffness matrices for wide side stimulation and [yy_thin_] and [kn_thin_] are the displacement and stiffness matrices for thin side stimulation. The vibrissa was divided into *n = 19* elements and a cubic displacement function is used to solve for the displacements and rotations ( respectively) of each element. An element is shaped as right rectangular prism where the functions *thin(x)* and *wide(x),* evaluated at the center of each element, determine depth and width of each element (these vary between elements but are constant in an element); the height of each element is the length of the vibrissa divided by n = 19. Since the vibrissa is fixed at its base, 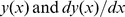 are set to zero in the first element of the FEM representing the base of the vibrissa. The FEM model was implemented in Mathematica™ and more details about it can be found in a previous study [3].

## Results

### The Fundamental Frequencies for the Two Modes of Motion

**Figure 5:**
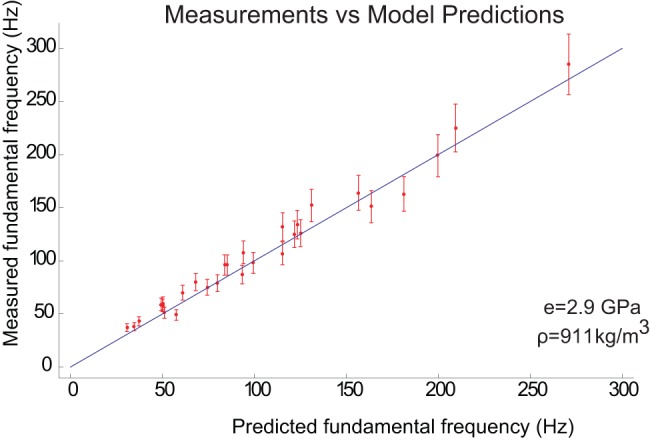
Shown with dots and error bars are the 29 measured fundamental frequencies for the two observed modes of motion for 14 vibrissae and for one observed mode of motion for the shortest vibrissa. Higher resonances than the fundamentals were difficult to detect and so were not measured.

Figure 5 illustrates the measured fundamental frequencies for the two modes of motion as a function of the models' predicted fundamental frequencies for all the fifteen vibrissae used. For the vibrissa whose length is 3.3 cm, only the fundamental frequency for the wide stimulation could be observed and so we have 29 data points. Repeated measurements of fundamental frequencies for four of the vibrissae, gave ∼10% differences and so 10% error bars are used in this figure and standard deviations of 10% of the measured frequencies were used in a chi square test of the model's predicted frequencies:
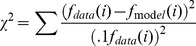



A Young's modulus of 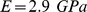 was used because that produced the lowest chi square test value (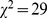). The chi square test for our 29 data points would have 28 degrees of freedom since we are only fitting one parameter (the Young's modulus) and therefore, since the chi square test value is about the same as the number of degrees of freedom of the chi square test, the model performs well in predicting the data. A Young's modulus of 2.9 GPa is plausible considering that we measured a Young's modulus range of 1.8–3.3 GPa on a 6 cm long seal vibrissa with an Instron 8845 Microtester™ at the MIT NanoMechanical Technology Laboratory; and the values found for rat whiskers were 3–4 GPa [2] and 7.8 GPa [1]). From the exact solution for the resonant frequencies of a thin elastic beam [10] we know that the predicted frequencies vary as the square root of the Young's modulus, and therefore errors in the predicted frequencies would vary as the square root of the errors in the Young's modulus.

### The Motion of the Vibrissae in Air

**Figure 6:**
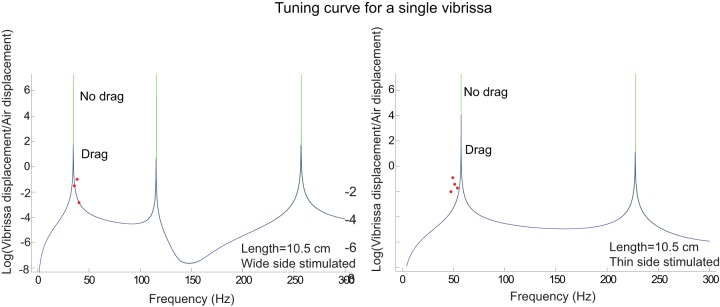
The tuning curves for the longest vibrissa (10.5 cm long) predicted by the model are presented for wide side stimulation (left panel) and thin side stimulation (right panel) which illustrate the general shape of the tuning curves for the fundamental, first and second harmonics for all the vibrissae. The y-axis of the plot shows the natural logarithm of the ratio of the maximum vibrissa displacement to the maximum air displacement. (The air displacement is approximated as half that of the speaker displacement). The green curves are the predicted motion without the drag term and the blue curves are the predicted motion with the drag term. The points represent the measured results. We see that the modes corresponding to the thin side being stimulated are at higher frequencies than the corresponding modes for the wide side being stimulated. We also note that the only effect of drag in air is to reduce the motion at the resonant frequencies. It should also be noted that the model has the most significant errors at the resonant frequencies where its predictions for the amplitudes of motion are usually much larger than the actual amplitudes.

Figure 6 illustrates the general shape of the tuning curves, on a log-linear scale, showing the fundamental frequencies as well as the first and second harmonics and also shows measurements near the fundamental frequencies where motion of the vibrissa was sufficiently large to be detected. As had been previously mentioned, motion was recorded at the fundamental resonant frequency, at the highest and lowest frequencies for which motion was observable, and for points in between those frequencies, if the range wasn't too small (less than ∼10% of the resonant frequency). We see that the tuning curves for the wide side being stimulated have correspondingly lower resonant frequencies than when the thin side is stimulated. The green curves represent the model predictions with no drag term and the blue curves are the model predictions with a drag term. By comparing the curves, we see that the effect of drag on the vibrissae, in air, is most important near the resonant frequencies, where the amplitude is largest, and that the model underestimates its effect. Another factor that could cause an error in the measurements at resonance is that the speaker may not be vibrating at a sufficiently precise frequency to see the peak in the amplitude. This imprecision was confirmed with signal generator and speaker frequency measurements.

**Figure 7:**
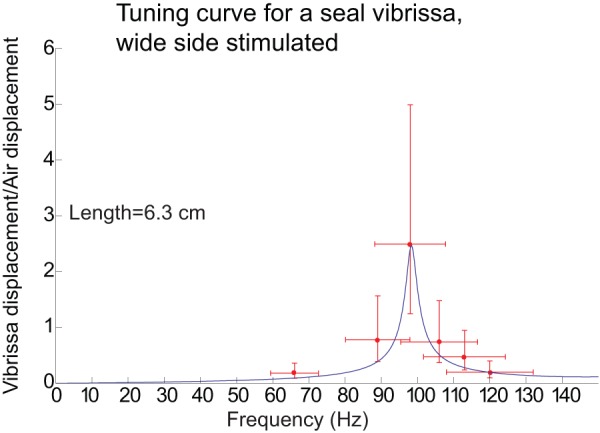
Tuning curve for the case where the model's predictions best matched with the data. The y-axis of demonstrates the ratio of the maximum vibrissa displacement to the maximum air displacement. The points represent a single displacement measurement at a particular frequency with 10% error bars for the frequency measurements (x-axis) and a factor of two (100% and 50%) for displacement measurements. The error bars derive from redoing the experiment where the vibrissa is not in the exact same position in front of the speaker as when the measurements were taken originally. All of the model predictions fall within the error bars of the data.

Figure 7 shows a tuning curve on a linear-linear scale where there was an excellent match between the data and the model, and the data falls on or close to the model's tuning curve, and well within the error bars, determined by repeating the experiment at another time, when the vibrissa was not in the exact same location in front of the speaker as before. This large variation in amplitude is likely due to the large variation in air displacements due to non-uniform shape of the speaker; and the curved shape of the vibrissa which would result in varying magnitudes of air displacements along the vibrissae; this was confirmed in the output of a velocity microphone in the vicinity of the vibrissa. For all the predicted results 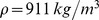 and 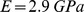.

**Figure 8:**
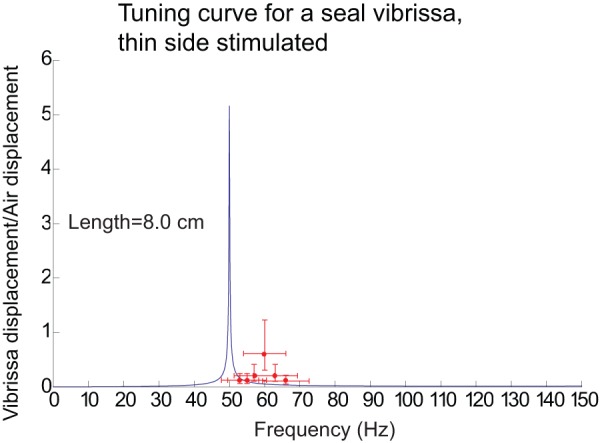
Tuning curve for the case where the model's predictions had a weaker match with the data. In this curve, the predicted tuning curve is about 10% lower than the measured tuning curve, and the model's prediction for the motion at resonance is larger than the actual measurement. The model predictions would fall within the error bars of the data if the model tuning curve were shifted to the right by 10. See Figure 7 for a description of the error bars.

Figure 8 shows a tuning curve where there was weaker match between the data and the model. Although the measurement frequencies do not fall on the predicted tuning curve, they are only about 10% lower than the measured frequencies, and if the curve were shifted over by 10%, the measurements would fall on the predicted tuning curve except at the fundamental resonant frequency, where the model's prediction is larger than the actual measurement. As had been previously mentioned, since the predicted curve is so narrowly tuned, this large error is likely due, in part, to the speaker's inability to vibrate at precisely the fundamental frequency. Another factor may be the twisting and curving of the vibrissa, which were not modeled, which would prevent the vibration mode from being maximally stimulated all along the vibrissa, and so the actual motion could be less than the predicted motion. For instance, the wide or thin side may only be directly facing the speaker over a fraction of the vibrissa's length and so the motion and its fundamental frequency may be smaller than what is predicted by the model. (The model assumes a straight vibrissa whose wide or thin side directly faces the speaker throughout the length of the vibrissa.) A third factor may be that, the drag term is derived from the motion of a cylinder whereas the vibrissae are rectangular in shape and so would have increased drag.

**Figure 9:**
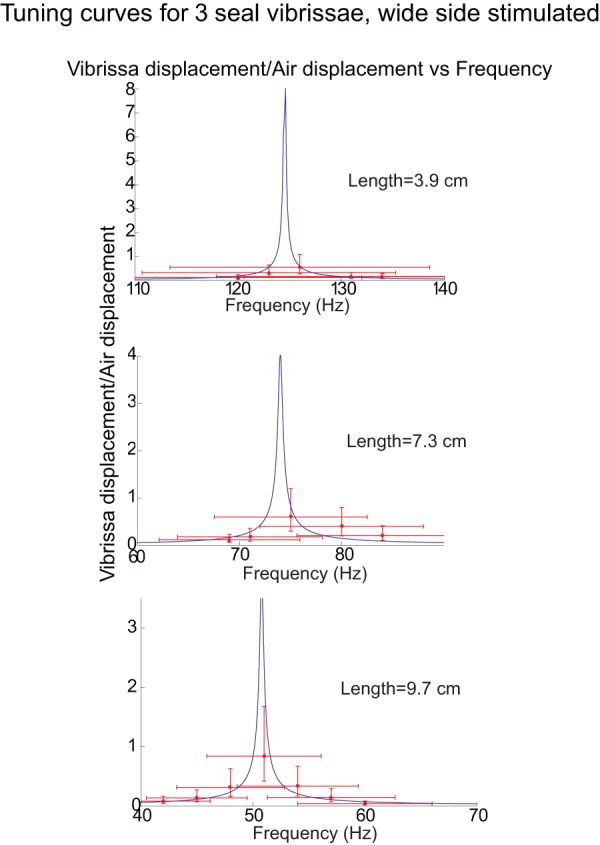
Motion of 3 vibrissae (short, medium, and long) for wide-side stimulation predicted by the model (curves) and measured (points). Each plot represents the tuning curve for a single vibrissa. The y-axis of each plot demonstrates the ratio of the maximum vibrissa displacement to the maximum air displacement. See Figure 7 for a description of the error bars. It can be observed that most of the model predictions fall within, or slightly outside, the error bars.

**Figure 10:**
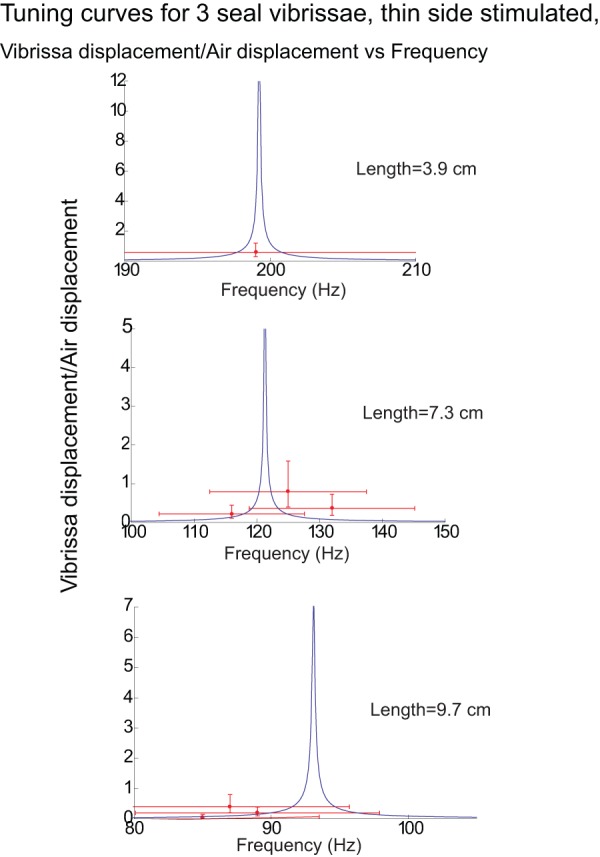
Motion of 3 vibrissae shown in **Figure 8** for thin-side stimulation predicted by the model (curves) and measured (points). Each plot represents the tuning curve for a single vibrissa. The y-axis of each plot demonstrates the ratio of the maximum vibrissa displacement to the maximum air displacement. See Figure 7 for a description of the error bars. It can be observed that most of the model predictions fall within, or slightly outside, the error bars. Motion of the vibrissae at frequencies outside of the range of shown was too small to measure.

Figure 9 and Figure 10 illustrate the measured and predicted motion of the vibrissae as a function of frequency for a representative sample of the 15 vibrissa, on a linear-linear scale. The model does well at predicting the motion of the vibrissae, with most of the predictions falling within, or slightly outside, the error bars, although it often overestimates the motion at resonance likely for the reasons previous mentioned in the discussion of Figure 8. As also discussed in Figure 8, the fundamental frequencies fall mainly within ±10% of the predicted frequencies.

#### The phase of the motion

To further confirm the accuracy of the model, we compared the phase of the measured motion to the model's predictions. The phase of the motion was measured using an analog video camera whose shutter speed could be increased so that the vibrissa was nearly stationary during a snapshot. Since the source was not synchronized to the camera, the phase of the motion could not be observed except at frequencies that are nearly integer multiples of the frame rate (29.97 Hz), where the strobe effect of the video camera served to “slow” the motion, and the phase difference θ between the speaker and the vibrissa could be observed.

**Figure 11:**
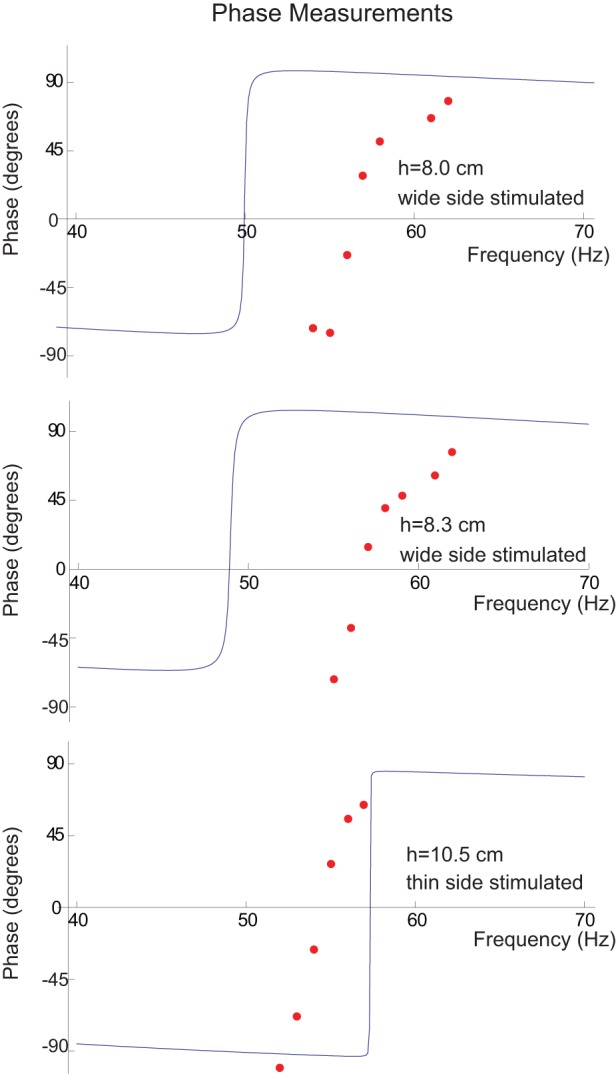
Phase measurements and model's predictions for three vibrissae with fundamental frequencies near 60 Hz. The strobe effect of the video camera was used to “slow” the motion so that phase differences could be observed. Much of the discrepancies between the location of the fundamental frequencies between measurements and the model are due to the ±10% errors in fundamental frequencies that had been seen with repeated measurements as had in discussed previously.

**Figure 12:**
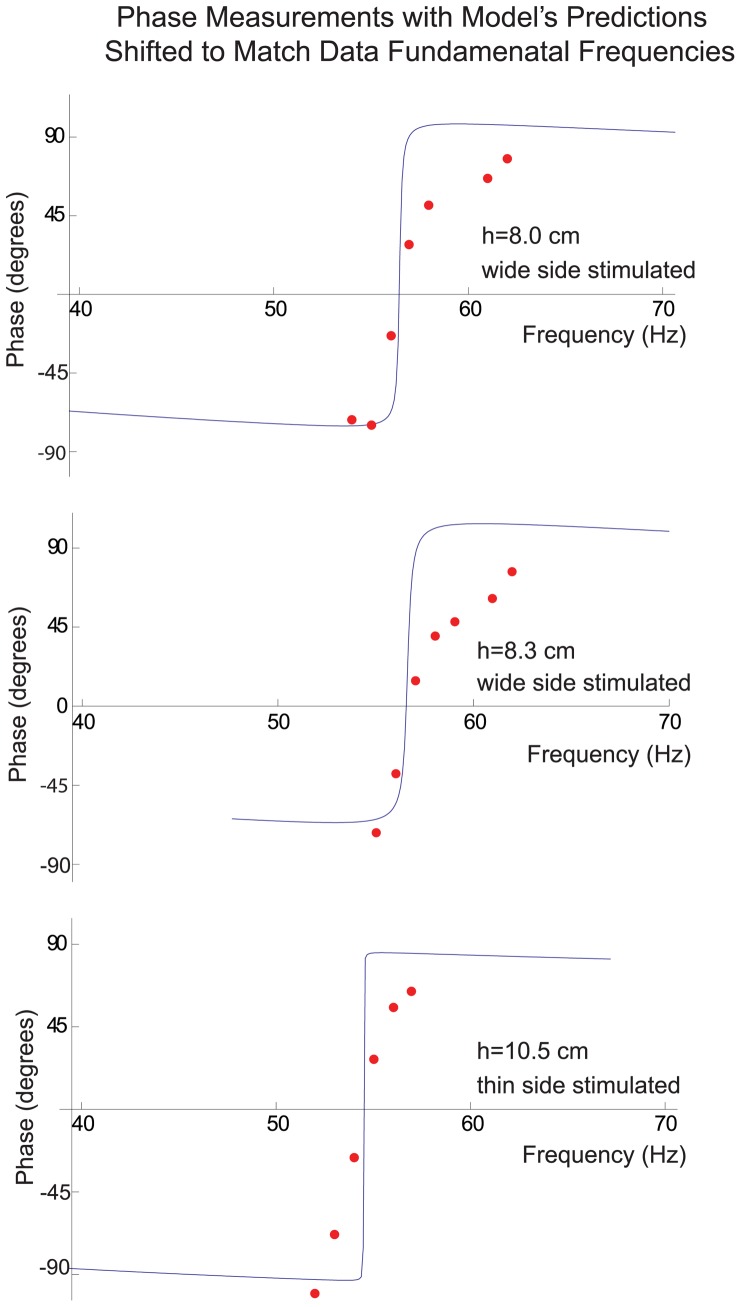
Phase measurements of the previous figure with model's predictions shifted to match the fundamental frequency of the data so that the comparison in the phase shifts between data and model can more easily be observed. From these curves we see that although the data indicate a phase change of nearly 180° for the motion before and the motion after resonance, the phase shift of the data seems to occur over a wider frequency range, a finding that is consistent with the motion data which show broader tuning than the model.

Figure 11 illustrates the measurements and the model's predictions for phase for an 8 cm long vibrissa whose wide side was stimulated with a measured fundamental frequency of 57 Hz, for an 8.3 cm long vibrissa whose wide side was stimulated whose measured fundamental frequency was also 57 Hz, and for a 10.5 cm long vibrissa whose thin side was stimulated, with a measured fundamental frequency was 55 Hz. Just as in the model, the vibrissa motion lags the speaker motion for input frequencies less than the fundamental frequency, is in phase at when the input frequency equals the fundamental, and then leads the speaker motion for frequencies beyond the fundamental. Much of the discrepancies between the location of the fundamental frequencies between measurements and the model are due to the ±10% errors in fundamental frequencies that had been seen with repeated measurements as has been discussed previously. To obtain a better comparison between the data and the model, the model's predictions are shifted to match the fundamental frequency of the data (Figure 12). Although the data indicate a phase change of nearly 180^o^ for the motion before and after resonance, the phase shift of the data seems to occur over a wider frequency range, which is consistent with the tuning curve data, which show broader tuning than the model.

### The Predicted Motion in Water

**Figure 13:**
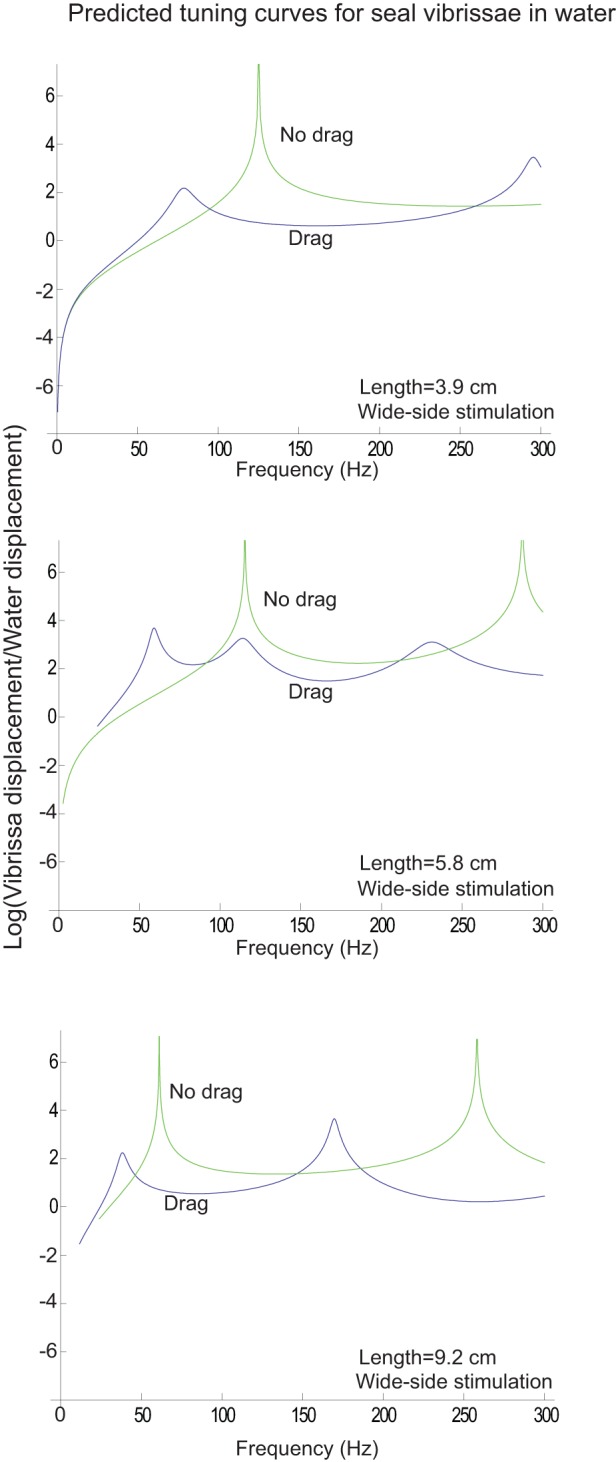
Predicted motion for three seal vibrissae in water. The y-axis of each plot shows the natural logarithm of the ratio of the maximum vibrissa displacement to the maximum water displacement. The lighter-shaded curves show the results when drag is not considered and the darker-shaded curves show the results when drag is considered. It is clear that drag in water not only broadens the tuning, but lowers the best frequencies. The predicted curves for thin-side stimulation are similarly shaped, with the resonances frequencies shifted to higher frequencies.

The equipment needed to measure the motion of the vibrissae in water proved to be beyond the means of this study and so shown in Figure 13 are the predicted curves for motion of the vibrissae in water for the case where drag is not considered (green curves) and where drag is considered (blue curves).

If the model is correct, it is clear that drag in water has a much more profound effect on vibrissa motion than in air. In water, we see that the drag lowers the positions of the resonant frequencies and broadens the tuning. Table 1 compares the fundamental frequencies in water with drag with fundamental frequencies with no drag (which are the same as the resonant frequencies in air). Without drag, the range of fundamental frequencies is 33 Hz-305 Hz. With drag in water, it is 20 Hz-200 Hz. The quality factor Q for the vibrissae is defined as:
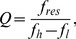
(6)where *f_res_* is the resonant frequency of the vibrissa, *f_h_* and *f_l_* are the frequencies above and below the resonance for which the amplitude is a factor of two smaller than at resonance. The quality factors of the vibrissae in air are predicted by the model to be on the order of 100 (Figures 9 and 10) although the measured tuning curves give a quality factor closer to 12 (Table 2). From Figure 13, we see that the quality factors of the vibrissae in water are predicted by the model to be about 6.

**Table 1:** Model predictions for the fundamental frequencies (Hz) in air and in water.

			Wide side stimulation		Thin side stimulation	
Length	Base cross-section (mm^2^)	Tip cross-section (mm^2^)	f1 (air)	f1 (water)	f1 (air)	f1 (water)
3.3 cm	.22	.008	271	163	303	193
3.9 cm	.22	.034	125	80	200	130
4.5 cm	.35	.048	164	98	209	134
5.8 cm	.43	.102	115	59	157	94
6.3 cm	.48	.057	99	75	181	80
6.9 cm	.51	.18	68	35	123	75
7.3 cm	.53	.23	74	38	122	73
7.7 cm	.56	.29	80	44	115	72
8.0 cm	.52	.26	50	29	84	53
8.3 cm	.57	.24	49	25	85	51
9.2 cm	.74	.045	33	18	61	40
9.7 cm	.77	.43	51	28	93	58
10.5 cm	.79	.47	35	20	58	37

**Table 2:** The quality factor Q for seal vibrissae and rat vibrissae in air.

Seal vibrissa			Rat vibrissa	
Length	Q (wide stimulation)	Q (thin stimulation)	Length	Q
3.9 cm	16	-	3.0 cm	4
4.5 cm	17	-	3.7 cm	5
5.8 cm	10	18	4.3 cm	2
6.3 cm	8	6		
6.9 cm	7	-		
7.3 cm	7.5	6		
7.7 cm	7	7		
8.0 cm	15	12		
8.3 cm	18	20		
9.2 cm	13	21		
9.7 cm	11	27		
10.5 cm	5	11		
Mean	11.8	12.9	3.7	

Q is about three times larger for seal vibrissae than for rat vibrissae.

## Discussion

### The Effect of Clamping on Vibrissae Tuning

We have just shown that the seal vibrissae that are clamped at their bases are tuned to frequencies in the range in water between 20 and 200 Hz. However, in vivo, the range of best frequencies will likely differ from this range since the level of attachment of the vibrissae at the follicle may not be as tight as clamped. How would the level of attachment affect the tuning properties of the vibrissae? For a simple elastic beam, the fundamental frequencies when it is free to rotate at its base, and free to displace and rotate at its tip are a factor of two higher than when it is clamped (cannot rotate) at its base and free to move (displace and rotate) at its tip [10]. This suggests that the in vivo frequency range is likely to be shifted higher but not by more than a factor of two.

### Comparison to Rat Vibrissae

**Figure 14:**
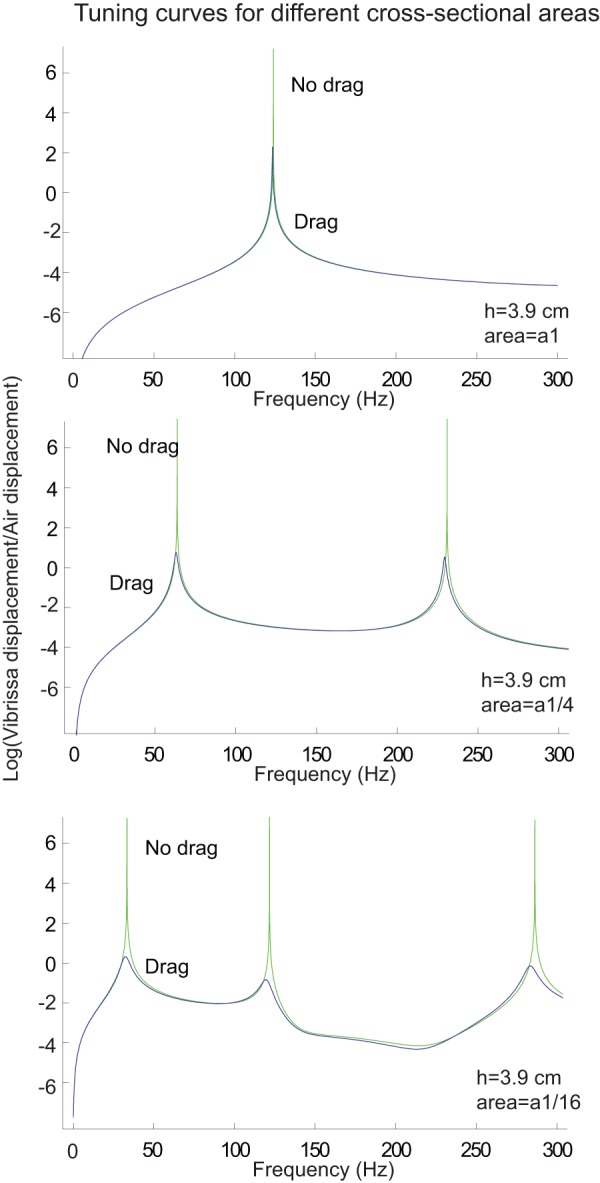
The change in tuning curves with decreasing cross-sectional areas for a seal vibrissa of length 3.9 cm (wide-side stimulation). The y-axis of each plot demonstrates the natural logarithm of the ratio of the vibrissa displacement to the air displacement. The top panel is the predicted tuning curve for the actual vibrissa dimensions. It is clear that the tuning shifts to lower frequencies and broadens as the cross sectional area of the vibrissa decreases. Similar results occur for thin-side stimulation.

It is interesting to compare seal vibrissae to those of rats. One key difference that has been previously mentioned is that the cross sections of rat vibrissae are circular while those of seals are rectangular. Another difference is the size of the cross-sectional areas. Table 3 illustrates the cross sectional areas at the base and tip of seal vibrissae and rat vibrissae of similar lengths. It is clear the cross-sectional areas of the seal vibrissae much larger than those of rat vibrissae, by a factor of 10 at the base and a factor of 50 at the tip. The size of the cross-sectional area has a significant effect on a tuning curve. Figure 14 illustrates the model's predictions for a tuning curve for a seal vibrissa with length 3.9 cm for 3 different cross-sectional areas. It is clear that as the cross-sectional area decreases, the resonant frequencies decrease and the tuning becomes broader. This observation makes sense– for a thin beam, the resonant frequencies are proportional to the radius *r*,
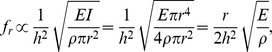

[10], and as the cross-sectional area decreases, the ratio of surface area to volume increases and therefore the effect of drag increases, broadening the tuning curve at the resonant frequencies.

**Table 3:** Comparison of seal and rat vibrissae cross-sectional areas.

Seal vibrissa length (cm)	Seal vibrissa base area (.1 mm^2^)	Seal vibrissa tip area (.1 mm^2^)	Rat vibrissa length (cm)	Rat vibrissa base area (.1 mm^2^)	Rat vibrissa tip area (.1 mm^2^)
3.3	2.2	0.08	3	0.24	0.00088
3.6	2.1	0.066	3.6	0.24	0.0039
3.9	2.2	0.34	3.6	0.24	0.0046
4.5	3.5	0.48	4.3	0.146	0.001
Averages					
3.825	2.495	0.14325	3.625	0.2165	0.002595

**Figure 15:**
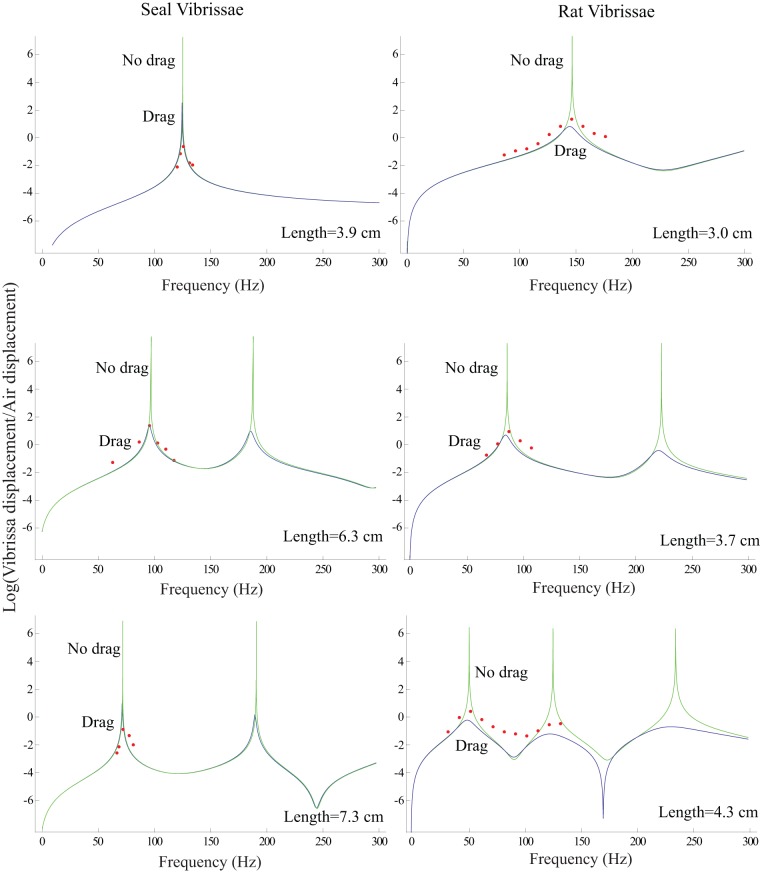
Tuning curves for seal vibrissae (wide side stimulation) and those for rat vibrissae. The y-axis of each plot shows the natural logarithm of the ratio of the vibrissa displacement to the air displacement. It is clear that the seal vibrissae give more sharply-tuned curves.

Figure 15 illustrates tuning curves for three seal vibrissae and three rat vibrissae and Table 2 illustrates the Q's for seal vibrissae and rat vibrissae. Since the quality factor Q, which is a measure of the selectivity of a system, is the same for when the wider side of the vibrissa is stimulated as when the thinner side is stimulated, only the tuning curve for the former stimulation is given. It is clear that the tuning is sharper for seal vibrissae than for rat vibrissae, with, when measured in air, 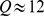 for the former and 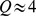 for the latter.

**Figure 16:**
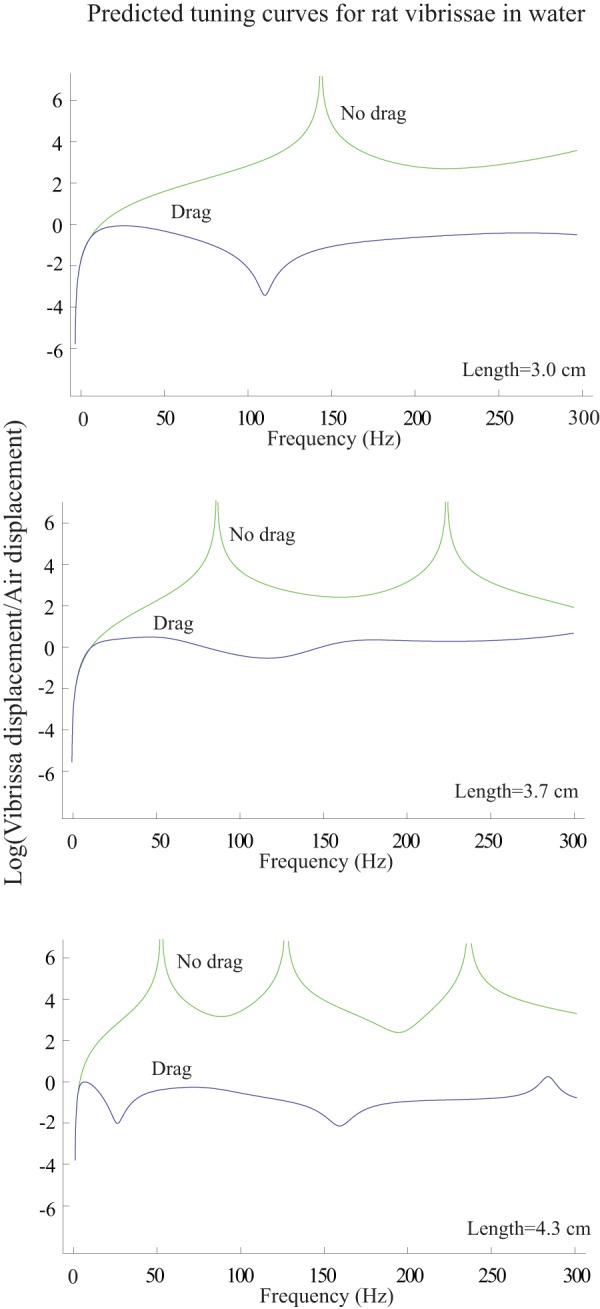
Predicted tuning curves for rat vibrissae in water. The y-axis of each plot demonstrates the natural logarithm of the ratio of the maximum vibrissa displacement to the maximum water displacement. Tuning is less observable for rat vibrissae in water than it is for seal vibrissae in water (Figure 13).

It is interesting to study the how the tuning curve of the rat vibrissae would change in water because we observed in Figure 13 that the drag due to water has the effect of both lowering the resonances and broadening the curves. Figure 16 illustrates predicted tuning curves in water for three rat vibrissae. As in the case of the seal vibrissae, the tuning for rat vibrissae is broadened in water, but because the tuning was less sharp than that of seal vibrissae in air, it is much less observable than for the seal vibrissae in water. This observation suggests a possible reason why the shapes of the cross sections of the vibrissae of seals and rats might differ. Seals, which spend most of the time in water, may depend on the tuning features of their vibrissae to sense their surrounding environment. For rats, the tuning properties of their vibrissae in water may be less critical.

### Vibrissae Tuning and Seal Behavior

The tuning properties of seal vibrissae that have been shown in this paper likely aid the seal in tracking the wakes of his prey. A recent study [14] found that the undulating morphology of the vibrissa, (not modeled in this study) prevented self-induced vibrations due to its vortex shedding as it is dragged through water. This feature allows the vibrissa, which is kept in an abducted position, to more readily sense the vibrations caused by external water disturbances such from as prey fish or other seals. Another recent study [8] looked at how well seals can distinguish between the wakes of objects with different shapes and sizes. Building on previous studies [7], [15], [16] that showed that harbor seals can track different types of hydrodynamic trails with their vibrissae, this recent study [8] showed that harbor seals, when their eyes and ears are covered so that their only means of sensing were their vibrissae, could distinguish between wakes made by paddles of different sizes and shapes, that were swept at random speeds. Each wake would have a particular frequency composition of water motion, which could be detected by a vibrissa system composed of vibrissae tuned to different frequencies. Moreover, the rectangular cross-section of the vibrissae provides extra modes of resonances and therefore may enhance the seal ability to analyze the water motion. A previous study [17] showed that the wakes of swimming fish can contain frequencies up to at least 100 Hz caused by a ladder like arrangement of vortices. Moreover a study [16] showed that blind-folded and ear-covered harbor seals can detect sinusoidally oscillating water movement in the 10–100 Hz frequency range. We determined that the range in water of fundamental resonant frequencies for seal vibrissae in vitro that are clamped at their bases is 20–200 Hz, and that in vivo this range may be shifted by as much as a factor of two, to higher frequencies if the attachment of the vibrissa at its follicle is less rigid. However, in either case, the frequency ranges would overlap those shown to be in the wakes of swimming fish and those shown to be detected by seals, and so would indicate that it is plausible that seals use their vibrissae to sense the wakes of their prey.

### Conclusion

The vibrissae of harp seals with lengths that range from 3.3 cm to 10.5 cm have been studied. These vibrissae have been found to have a mass density of 911 kg/m^3^ and to have tapered rectangular cross-sections, unlike those of rats which have tapered round cross-sections. Their cross sections were also observed to be much wider than those of rat vibrissae. The vibrissae were stimulated by sound in air and two modes of motion were observed – one corresponding to the wider side being stimulated (when the wider side faced the speaker) and the other corresponding to the thinner side (when it faced the speaker). The fundamental frequencies for the two modes were measured as well as the motion of the vibrissae that was observable at frequencies near the fundamental frequencies. The fundamental frequencies and the motion of the vibrissae matched those predicted by an FEM model, using a Young's modulus of 2.9 GPa.

The tuning curves of the seal vibrissae in air had quality factors of ∼12 and were sharper than those of rat vibrissae with quality factors of ∼4. Predicted tuning curves of seal vibrissae in water were less sharp – however tuning was still pronounced, which was not the case for predicted tuning curves of rat vibrissae in water. This observation suggests the reason for the difference in size and shape of the seal vibrissae from the rat vibrissae because seals would be more dependent on their vibrissae's motion in water than would be a rat.

The fundamental frequencies of the seal vibrissae were found to be in water between 20 and 200 Hz, although this range may be shifted up depending on the degree of attachment at the vibrissa follicle, yet we would not expect it to be shifted by more than a factor of two. Therefore the range of the fundamental frequencies overlap the range in which blind-folded and ear-covered seals have been shown to be sensitive, and the range of frequency components of the wakes of swimming fish which suggests that seals may use the resonances of their vibrissae for detection.
